# Genomic Evidence Supports the Recognition of Endometriosis as an Inflammatory Systemic Disease and Reveals Disease-Specific Therapeutic Potentials of Targeting Neutrophil Degranulation

**DOI:** 10.3389/fimmu.2022.758440

**Published:** 2022-03-23

**Authors:** Chaohui Bao, Hengru Wang, Hai Fang

**Affiliations:** ^1^ Shanghai Institute of Hematology, State Key Laboratory of Medical Genomics, National Research Center for Translational Medicine at Shanghai, Ruijin Hospital Affiliated to Shanghai Jiao Tong University School of Medicine, Shanghai, China; ^2^ Faculty of Medical Laboratory Science, Ruijin Hospital Affiliated to Shanghai Jiao Tong University School of Medicine, Shanghai, China

**Keywords:** endometriosis, inflammatory systemic disease, therapeutic target prioritization, drug repurposing, neutrophil degranulation

## Abstract

**Background:**

Endometriosis, classically viewed as a localized disease, is increasingly recognized as a systemic disease with multi-organ effects. This disease is highlighted by systemic inflammation in affected organs and by high comorbidity with immune-mediated diseases.

**Results:**

We provide genomic evidence to support the recognition of endometriosis as an inflammatory systemic disease. This was achieved through our genomics-led target prioritization, called ‘*END’*, that leverages the value of multi-layered genomic datasets (including genome-wide associations in disease, regulatory genomics, and protein interactome). Our prioritization recovered existing proof-of-concept therapeutic targeting in endometriosis and outperformed competing prioritization approaches (Open Targets and Naïve prioritization). Target genes at the leading prioritization revealed molecular hallmarks (and possibly the cellular basis as well) that are consistent with systemic disease manifestations. Pathway crosstalk-based attack analysis identified the critical gene *AKT1*. In the context of this gene, we further identified genes that are already targeted by licensed medications in other diseases, such as *ESR1*. Such analysis was supported by current interests targeting the PI3K/AKT/mTOR pathway in endometriosis and by the fact that therapeutic agents targeting *ESR1* are now under active clinical trials in disease. The construction of cross-disease prioritization map enabled the identification of shared and distinct targets between endometriosis and immune-mediated diseases. Shared target genes identified opportunities for repurposing existing immunomodulators, particularly ﻿disease-modifying anti-rheumatic drugs (such as *TNF*, *IL6* and *IL6R* blockades, and *JAK* inhibitors). Genes highly prioritized only in endometriosis revealed disease-specific therapeutic potentials of targeting neutrophil degranulation – the exocytosis that can facilitate metastasis-like spread to distant organs causing inflammatory-like microenvironments.

**Conclusion:**

Improved target prioritization, along with an atlas of *in silico* predicted targets and repurposed drugs (available at https://23verse.github.io/end), provides genomic insights into endometriosis, reveals disease-specific therapeutic potentials, and expands the existing theories on the origin of disease.

## Introduction

Endometriosis, characterized by the presence of ectopic endometrial-like tissues, is classically viewed as a chronic hormone dependent neuroinflammatory disease locally restricted to the pelvis (1). Endometriosis is estimated to be present in ﻿50–80% of women with pelvic pain, and occurs in up to 50% of women with infertility (2, 3). Clinical manifestations are varied, with high prevalence (affecting 5-10% of reproductive-age women with negative impacts on quality of life), common misdiagnosis (65% of women initially misdiagnosed) or diagnostic delay (delayed by 4-11 years). Currently, endometriosis is increasingly recognized as a systemic disease with multi-organ effects throughout the body [reviewed in (4)]. The full effects are far from clear, but consensus has been reached that patients with endometriosis are likely to have: (i) adipocyte and hepatic metabolic changes, such as low body mass index (5); (ii) neurological alterations that enhance pain sensitivity and increase the risk of developing mood disorders, such as fatigue, depression and anxiety (6); (iii) systemic inflammation that causes widespread inflammatory-like microenvironments in affected tissues or organs (7); and (iv) the tendency to develop immune-mediated diseases (8).

Likely due to the complexity of systemic effects and disease comorbidities described above, there is currently no cure for endometriosis. Available treatments help to reduce symptoms and maintain quality of life. Two treatment options (medical and surgical therapies) are mainly for alleviating endometriosis-associated pain (4). Medical therapy, combining oral contraceptives (or progestins) and non-steroidal anti-inflammatory drugs (known as ‘NSAIDs’), represents the first-line therapy. On average, 25-33% of patients do not respond to first-line therapies. For non-responders with persistent pain, gonadotropin-releasing hormone (GnRH) analogs or aromatase inhibitors represent the second-line therapy. Surgical therapy aims to treat pain and disease-related infertility as well. Medical therapy following surgery represents the third-line therapy that is supposed to reduce or minimize disease recurrence and systemic effects. It should be noted, however, that neither medical therapy nor surgery fully addresses the systemic nature of the disease. Recognizing endometriosis as a systemic disease highlights the importance of new target identification and validation to increase the range of medical therapies, ultimately for better therapeutics.

**Figure 1:**
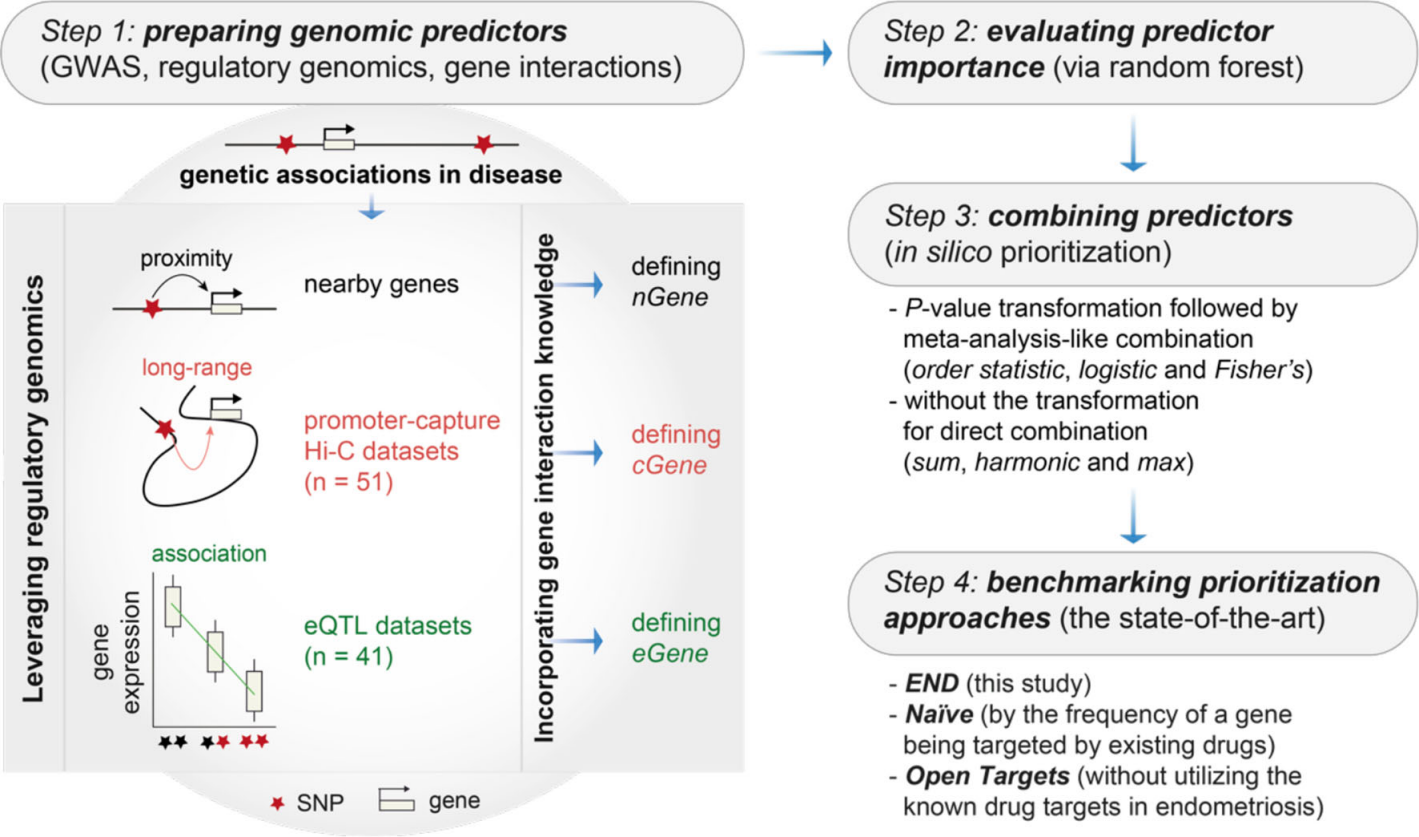
Overview of genomics-led target prioritization in endometriosis (called ‘*END*’). Steps 1-3 sequentially describe how to prepare, evaluate and combine genomic predictors for *in silico* target prioritization. The key is the preparation of genomic predictors at Step 1, as illustrated on the right, utilizing genetic associations in disease, regulatory genomics (mainly promoter-capture Hi-C datasets and eQTL datasets) and the knowledge of gene/protein interactions. Step 4 is intended for benchmarking to compare the performance of *END* with two competing prioritization approaches, including *Naïve* (a gene prioritized simply according to how often the gene is already targeted by existing drugs) and *Open Targets* (without utilizing the information of known drug targets). GWAS, genome-wide association studies; *nGene*, nearby genes; *cGene*, conformation genes; *eGene*, expression genes.

The etiopathogenesis of endometriosis is complex, involving the interplay between genetic inheritance and environmental influence. Genetic associations arising from genome-wide association studies (GWAS) provide a rich source of genetic targets. For example, GWAS meta-analysis in endometriosis has identified disease risk loci that are likely to affect genes involved in hormone metabolism (9). Such genetic evidence is critical for successful therapeutic development (10, 11). How to harness GWAS findings for use in drug discovery (12) and drug repurposing (13), however, requires a paradigm change in strategies. Recently, we have proposed a strategic framework that generalizes how to establish the link from genetic loci to modulated genes that can be further linked to drug targets (14, 15). Our advocates of genetic target prioritization have driven this field of research (16–22). In this study, we extended this into genomics-led target prioritization (called ‘*END’*) and demonstrated better performance than the *status quo* approaches (**Figure 1**), with the aim of providing genomic evidence for endometriosis as an inflammatory systemic disease. Our genomic prioritization, followed by a range of integrative bioinformatics analyses (including comparisons with immune diseases), enabled us to identify repurposing opportunities for existing immunomodulatory drugs and more importantly, to reveal therapeutic strategies for endometriosis-specific targeting. In a wider context, our study provides a new strategy to advance the use of human genetics and genomics for target identification and validation in inflammatory systemic diseases.

## Materials and Methods

### Genomics-Led Prioritization and Performance Evaluation

The steps designed for genomic prioritization and evaluation were briefly described in **Figure 1** and detailed below.

#### Step 1: Preparing Genomic Predictors

We extended our previous pipeline (23) to prepare genomic predictors utilizing multi-layered genomic datasets and the knowledge of gene/protein interactions. Genomic datasets included: (i) GWAS summary statistics in endometriosis (9, 24–27), considering a typical threshold (*P-*values < 5×10^−8^) and SNPs in linkage disequilibrium (R^2^<0.8) according to the European population (28) to define nearby genes (*nGene*); (ii) promoter capture Hi-C (29–34), defining conformation genes (*cGene*); and (iii) eQTL (35–41), defining expression genes (*eGene*). The knowledge of gene/protein interactions was obtained from the STRING database (high-quality, only with evidence codes ‘experiments’ or ‘databases’) (42), corresponding to a total of 14,325 target genes considered for prioritization (**Table S1**).

#### Step 2: Evaluating Predictor Importance

We applied random forest (43) to evaluate predictor importance. Considering the fact that the *nGene* predictor is conventionally used (thus treated as the baseline), only *cGene* and *eGene* predictors that were no less important/informative than *nGene* remained for the subsequent combination.

#### Step 3: Combining Predictors

We combined informative predictors for *in silico* prioritization. For a candidate target gene, strategies that combine individual predictors could be either direct or indirect. The direct combinations can be *sum* (summing up affinity scores), *max* (taking the maximum) or *harmonic* (using the harmonic sum), while the idea of indirect combinations is to first transform affinity scores into *P*-values and then combined *via* meta-analysis methods such as *Fisher’s*, *logistic* or *order statistic*. Performance for combination strategies was measured by the area under the ROC curve (AUC) separating clinical proof-of-concept targets in endometriosis from simulated controls. Existing proof-of-concept targets in endometriosis were defined as therapeutic target genes of drugs reaching development phase 2 and above [sourced from the ChEMBL database (44)].

#### Step 4: Benchmarking Prioritization Approaches

We benchmarked competing approaches for performance evaluation. Benchmarking was also based on AUC (that is, separating clinical proof-of-concept targets in endometriosis from simulated controls) to compare the performance of ‘*END*’ (this study), ‘*Naïve*’ (an approach prioritizing a gene by how often it has been targeted by existing drugs), and ‘*Open Targets*’ (using the harmonic sum to aggregate individual evidence except for the information on known drugs) (45).

### Target Set Enrichment Analysis

The dnet package (46) was used to conduct target set enrichment analysis quantifying the degree to which a predefined gene list is enriched at the leading prioritization. The leading prioritization, visually defined as the left-most region of the peak in running enrichment plot, is the core subset of the prioritized target genes accounting for the enrichment signal. The analysis was applied to clinical proof-of-concept targets in endometriosis, showing that they tended to be highly ranked. This analysis was also applied to: (i) cell-type-specific gene signatures (47) for exploring the cellular basis of all prioritized target genes in endometriosis; (ii) MSigDB hallmark gene sets (48) for revealing molecular hallmarks based on the top 10% prioritized target genes in endometriosis; and (iii) one fibroblast gene expression signature specific to ectopic endometrium (49) and four gene expression signatures involving two endometriosis stages (I/II and III/IV) at two menstrual cycle phases (proliferative and secretory) (50) for examining the highly prioritized target gene expression.

### Pathway Crosstalk-Based Attack Analysis

There were three steps to achieve this. First, the XGR package (51) and a collection of KEGG organismal system pathways (52) were used to identify enriched pathways based on the top 1% prioritized genes. Second, pathway crosstalk was identified by searching for a subset of gene interactions (extracted from enriched pathways identified in the previous step); identified so in a manner that the resulting pathway crosstalk contained highly prioritized and interconnected genes (53). Third, attack analysis was conducted to select optimal targeting combinations that maximized the effect of removing nodes on the crosstalk; done so in a manner to maximize the effect of removing either single nodes or specific node combinations. The combinatorial node removal can be 2-node combinations removed in the context of a fixed node ‘*ATK1*’, and 3-node combinations removed in the context of two fixed nodes ‘*AKT1* + *ESR1*’.

### Construction of Cross-Disease Prioritization Map

Focusing on the top 5% prioritized target genes (n = 689) in endometriosis, the supraHex package (54) was used to construct cross-disease prioritization map. In brief, a supra-hexagonal map, consisting of 91 hexagons, was trained by the prioritization matrix (containing priority ratings) of 689 target genes *versus* 7 diseases including endometriosis (this study) and 6 immune-mediated diseases (53). These immune diseases include: (i) inflammatory bowel diseases, subdivided into Crohn’s disease (CRO) and ulcerative colitis (UC); and (ii) inflammatory systemic diseases, including multiple sclerosis (MS), rheumatoid arthritis (RA), Sjögren’s syndrome (SJO), and systemic lupus erythematosus (SLE). The trained map was used to illustrate a gene prioritization profile per disease, and together with a consensus neighbour-joining tree built from the prioritization matrix, to further illustrate inter-disease relationships. The trained map was also divided into target gene clusters in a topology-preserving manner. Enrichment analysis for genes within a cluster was based on one-sided Fisher’s exact test to identify enrichments in terms of: (i) approved drug targets obtained from the ChEMBL database (44); (ii) immune system pathways from the Reactome database (55); (iii) Gene Ontology functional annotations from NCBI (56); and (iv) mouse phenotype annotations using Mammalian Phenotype Ontology (57).

### Identification of Druggable Pockets

The fpocket software (58) was used to predict druggable pockets of a target gene based on its known protein structure(s). The known protein structures were obtained from the PDB database (59), for example, the access code ‘4N78’ for the WAVE regulatory complex (60). A gene was defined to be tractable if predicted to have drug-like binding sites (that is, druggable pockets). The PDB structure was viewed in 3D as cartoon (secondary structure abstraction), colored by PDB chains and embedded with druggable pockets in blue (61).

## Results

### Leveraging Genomic Predictors for *In Silico* Prioritization of Therapeutic Targets in Endometriosis

**Figure 2:**
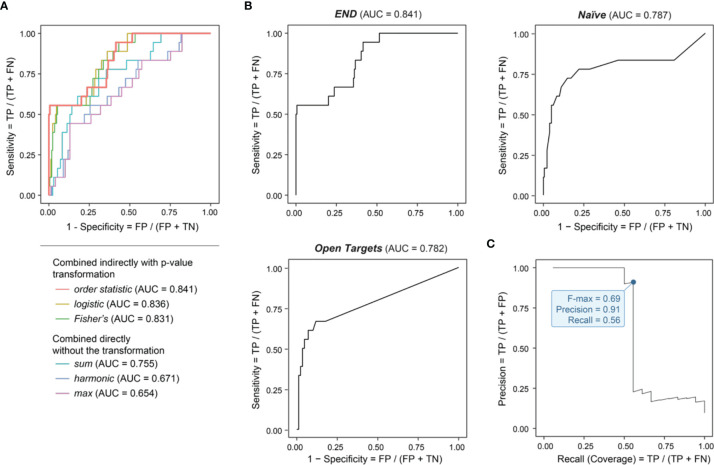
Genomic prioritization in endometriosis. **(A)** Comparisons of strategies combining predictors. The combination strategy based on *order statistic* achieves the optimal performance, measured by the area under the ROC curve (AUC) separating clinical proof-of-concept targets from simulated negative controls. FN, false negatives; FP, false positives; TN, true negatives; TP, true positives. **(B)** Benchmarking prioritization approaches as described in **Figure 1**, in terms of AUC. **(C)** Precision-Recall curve for *END*, with the F-max metric indicated (corresponding to 91% precision and 56% recall).

We performed genomics-led prioritization, taking GWAS summary statistics in endometriosis as inputs and leveraging the informativeness of regulatory genomics in diverse cell types, activation states and tissues. As outlined in **Figure 1** (see *Materials and Methods* for details), our multi-step prioritization process consisted of: (i) the preparation of genomic predictors; (ii) the evaluation of predictor importance to identify informative predictors; (iii) the assessment of how to combine informative predictors; and (iv) performance evaluation to benchmark competing approaches. We found that using the meta-analysis-like combination strategy, particularly based on *order statistic* to combine genomic predictors, achieved much better performance than using the direct combination strategy (**Figure 2A**). Benchmarking results showed that our prioritization approach (called ‘*END*’) was superior to competing approaches (**Figure 2B**). We considered two competing approaches, namely, *Open Targets* (also using genetics and genomics for target identification and prioritization) (45) and *Naïve* prioritization (prioritizing a gene by how often it has been targeted by existing drugs), that were similar in performance. Notably, *Naïve* prioritization, based on the concept of drug repurposing, was limited in being unable to predict new targets. The precision-recall analysis showed that our prioritization achieved the 91% precision at the 56% recall (prioritization coverage; **Figure 2C**).

**Figure 3:**
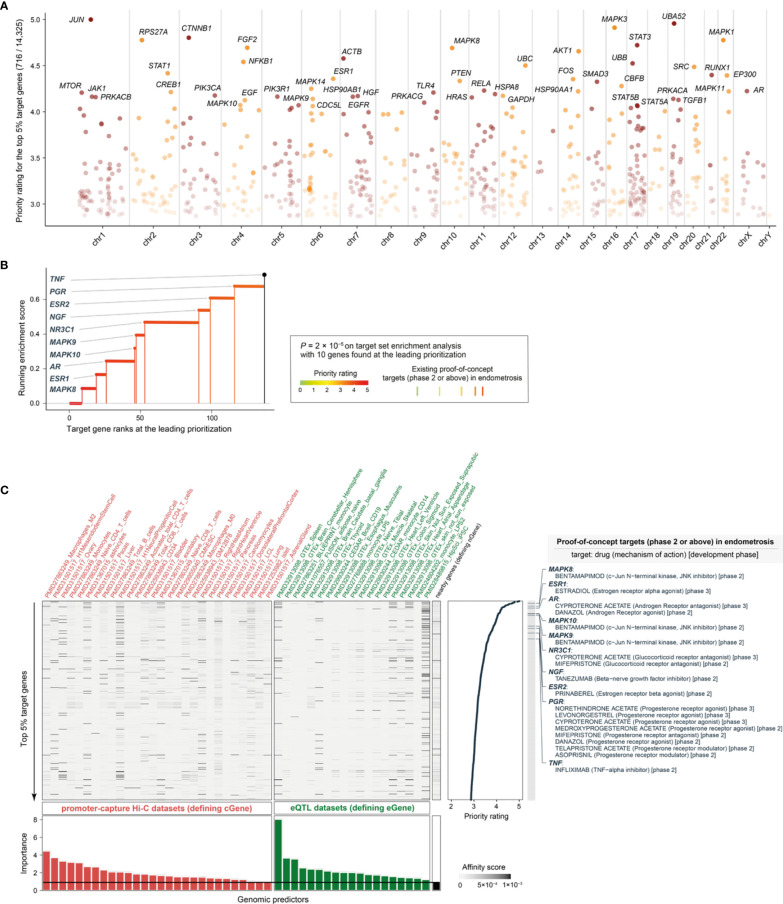
Performance evaluation for prioritization in endometriosis. **(A)** Manhattan plot illustrating priority rating (y-axis) for top 5% target genes across chromosomes (x-axis), with top 30 named. **(B)** Leading prioritization plot for clinical proof-of-concept targets in endometriosis, which are indicated in vertical lines (also color-coded by priority rating). **(C)** Heatmap plot illustrating predictor matrix used in *END*. Shown beneath is relative importance of predictors (color-coded by dataset types), quantified by the accuracy decrease (disabling a predictor), and the horizontal line indicates the importance for *nGene* (that is, the nearby gene predictor that is conventionally used and thus here treated as a baseline predictor). Shown on the right is the information of prioritized target genes (only the top 5% shown for simplicity), together with the information on existing proof-of-concept targets in endometriosis (including drugs, mechanisms of action, and development phases). eQTL, expression quantitative trait locus.

We prioritized a total of >14,000 candidate target genes [with the knowledge of gene interactions sourced from the STRING database (42)], ranked by priority rating (**Table S1**). The top prioritized targets included genes essential in inflammation and cellular responses, such as *JUN* (ranked 1^st^), *NFKB1* (12^th^), *RUNX1* (17^th^), *PTEN* (21^st^), *MAPK14* (24^th^), *FOS* (20^th^), *SMAD3* (22^nd^), and *RELA* (25^th^) (**Figure 3A**). Our prioritization recovered existing clinical proof-of-concept target genes in endometriosis (**Figures 3B, C**). These proof-of-concept targets in endometriosis were all at the top 1% prioritized gene list, including: *MAPK8* (9^th^), *MAPK10* (46^th^), and *MAPK9* (47^th^) targeted by bentamapimod, a JNK inhibitor; *ESR1* (19^th^) targeted by estradiol, an estrogen receptor (ER) alpha agonist; *ESR2* (99^th^) targeted by prinaberel, an ER beta agonist; *AR* (26^th^) targeted by danazol, an androgen receptor (AR) agonist, and cyproterone acetate, an AR antagonist; *NR3C1* (53^th^) targeted by cyproterone acetate and mifepristone, two glucocorticoid receptor antagonists; *NGF* (91^th^) targeted by tanezumab, a beta-nerve growth factor inhibitor; *PGR* (116^th^) targeted by selective progesterone receptor modulators (SPRMs); and *TNF* (136^th^) targeted by infliximab, a TNF-alpha inhibitor. According to the latest ESHIR guideline (62), SPRMs such as medroxyprogesterone acetate and levonorgestrel are highly recommended to reduce endometriosis-associated pain, and norethindrone acetate (alongside GnRH) highly recommended to prevent bone loss and hypoestrogenic symptom, but no longer recommended for danazol. **Figure 3C** also provides a summary of the predictors used and their relative importance (informativeness). We treated the nearby gene predictor as the baseline defining predictive informativeness. We identified informative regulatory genomic predictors that were mostly derived from immune blood cells (with a few from other cell types and tissues). These results necessitated the use of genomic datasets from diverse contexts for target prioritization and identification, which is consistent with endometriosis that is increasingly recognized as a systemic disease (4).

### Characterizing the Cellular Basis and Molecular Hallmarks of Target Gene Prioritization in Endometriosis

**Figure 4:**
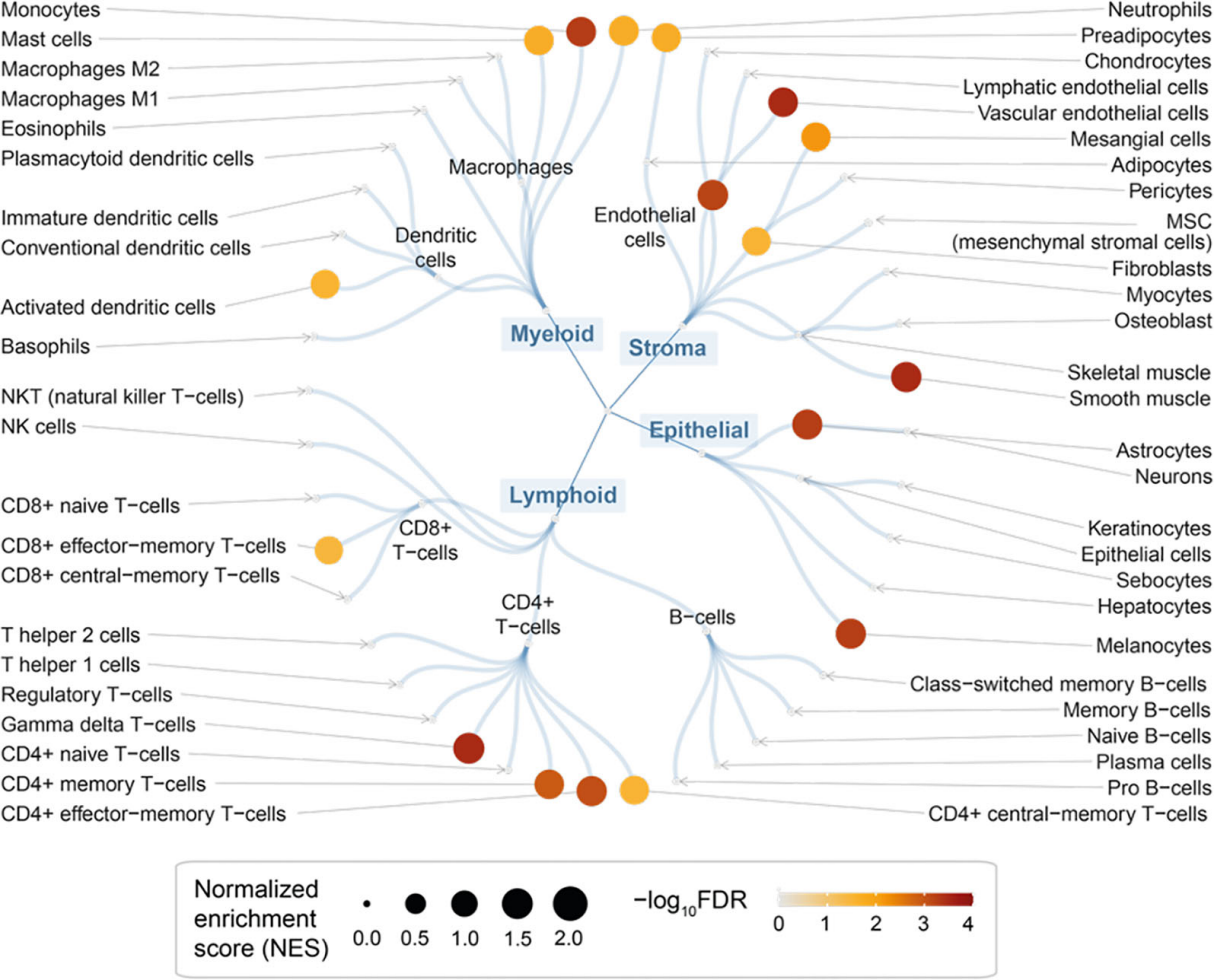
Cellular basis of therapeutic targeting in endometriosis. Circular overview of cell type enrichments, with nodes sized by the normalized enrichment score (NES) and colored by the enrichment significance (FDR) calculated using target set enrichment analysis.

We used rank-based target set enrichment analysis to characterize prioritized target genes using cell-type-specific gene signatures (47). We found the enrichment for immune cells; these included cells in myeloid lineages (such as monocytes, neutrophils, and mast cells) and lymphoid lineages (such as gamma delta T-cells, CD4 + memory T-cells, and CD8+ effector-memory T-cells) but not in B-cell lineages (**Figure 4** and **Table S2**). In addition to immune cells (for example, gamma delta T-cells; **Figure S1A**), we also found the enrichment for stromal cells (**Figure S1B**) and epithelial cells (**Figure S1C**). Taken together, our findings are consistent with evidence of altered immunity and inflammation (local and systemic) in endometriosis (3), and also reveal the cellular basis that might involve multiple lineages underlying the pathogenesis of endometriosis.

**Figure 5:**
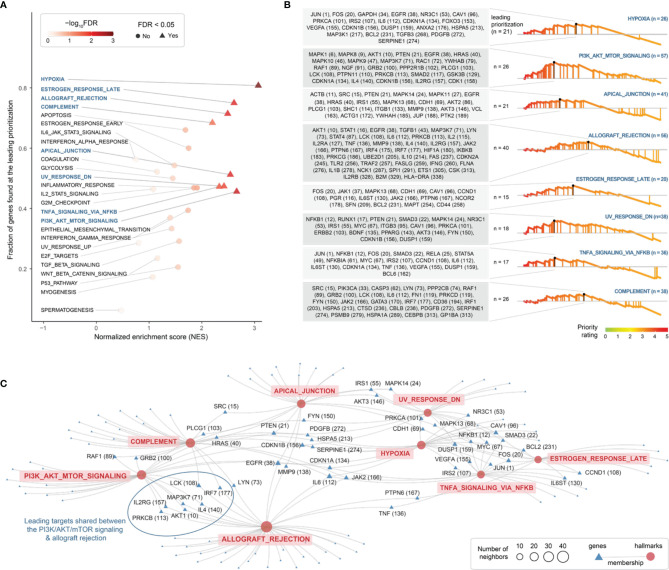
Exploring molecular hallmarks of therapeutic targeting in endometriosis. **(A)** Scatter plot of molecular hallmark enrichments, including normalized enrichment score (NES; x-axis), fraction of hallmark genes found at the leading prioritization (y-axis), and the enrichment significance (FDR) calculated using target set enrichment analysis. Enriched hallmarks (FDR < 0.05) are shaped in triangle and labelled in blue. **(B)** Details on enriched hallmarks. *Left*: the gene list found at the leading prioritization, with the number in the parentheses indicating priority rank. *Right*: the leading prioritization (visually defined as the left-most region ahead of the peak). **(C)** Network-like representation of enriched hallmarks and member genes. Inter-hallmark sharing genes are also labelled including priority rank. For example, genes shared between two hallmarks (the PI3K/AKT/mTOR signalling and allograft rejection) are framed in oval shape.

Next, using MSigDB hallmark gene sets (48), we identified 8 molecular hallmarks enriched in highly prioritized genes (**Figure 5A** and **Table S3**). These included: cellular responses to low oxygen levels (hypoxia), ultraviolet radiation and estrogen; signaling pathways (NF-kB signaling in response to TNF, and activation of the PI3K/AKT/mTOR pathway); immune processes (allograft/transplant rejection, and the complement system); and the apical junction complex. For each of these hallmarks, genes found at the leading prioritization are shown in **Figure 5B**. We identified 21 leading prioritized genes that are responsive to hypoxia, with 4 genes (*HSPA5*, *IL6*, *PDGFB*, and *SERPINE1*) encoding components of the complement system and 7 genes (*CDKN1A*, *DUSP1*, *FOS*, *IL6*, *IRS2*, *JUN*, and *VEGFA*) regulated by NF-kB in response to TNF (**Figure 5C**). We identified 26 leading prioritized genes involved in the PI3K/AKT/mTOR pathway (**Figure 5B**), the pathway relevant to the severity of endometriosis stages (63). Among these 26 genes, 7 (*AKT1*, *EGFR*, *IL2RG*, *IL4*, *LCK*, *MAP3K7*, and *PRKCB*) are also involved in allograft rejection (**Figure 5C**), the consequences of which may cause tissue injury and fibrosis. These results revealed diverse but related molecular hallmarks in endometriosis, indictive of being an inflammatory systemic disease.

### Pathway Crosstalk-Based Attack Analysis Identifies Critical Genes and Repurposed Drugs

**Figure 6:**
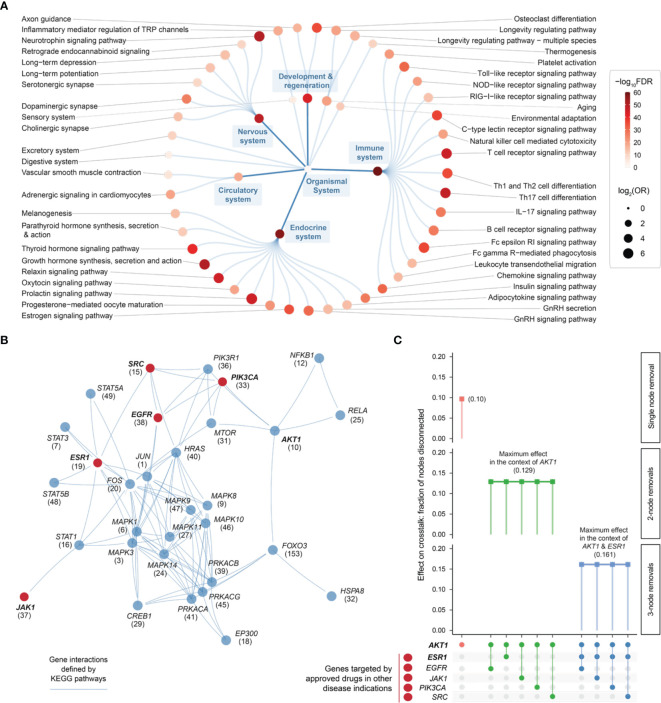
Pathway crosstalk-based attack analysis identifies critical genes and repurposes licensed medications. **(A)** Pathway enrichments of the top 1% prioritized target genes. Based on KEGG organismal system pathways that are organized circularly. Each dot represents a pathway, sized by the odd ratio (OR) and colored by the significance level (FDR) calculated using one-sided Fisher’s exact test. **(B)** Pathway crosstalk identified by integrating target prioritization information with gene interactions (extracted from enriched pathways). Nodes are labelled by gene symbols (priority rank) and highlighted in red if currently targeted by approved drugs in other disease indications. **(C)** Effect of node removals on the crosstalk. Top: the single-node effect maximized by removing *ATK1*, the maximum effect of removing another approved drug target in the context of *AKT1*, and the maximum effect of removing another approved drug target in the context of *AKT1* and *ESR1*. Bottom: node removals are indicated by colored circles.

Using the KEGG resource (52), we proceeded to perform pathway enrichment analysis for the top 1% prioritized target genes. This identified a wide range of organismal system pathways that can be broadly categorized into immune, endocrine, nervous, and reproductive systems (**Figure 6A**). It is well-recognized that maladaptation of these organismal systems can occur in chronic inflammatory systemic diseases (64). Integrated analysis of these enriched pathways identified a 31-gene network (*P* = 3.1 × 10^−213^ on permutation test), reflective of crosstalk between pathways, that all contained highly prioritized genes in endometriosis (**Figure 6B** and **Table S4**). Using the information on approved therapeutics available in the ChEMBL database (44), we found 5 genes that are already targeted by licensed medications (approved drugs) in other diseases (**Figure 6B**).

**Figure 7:**
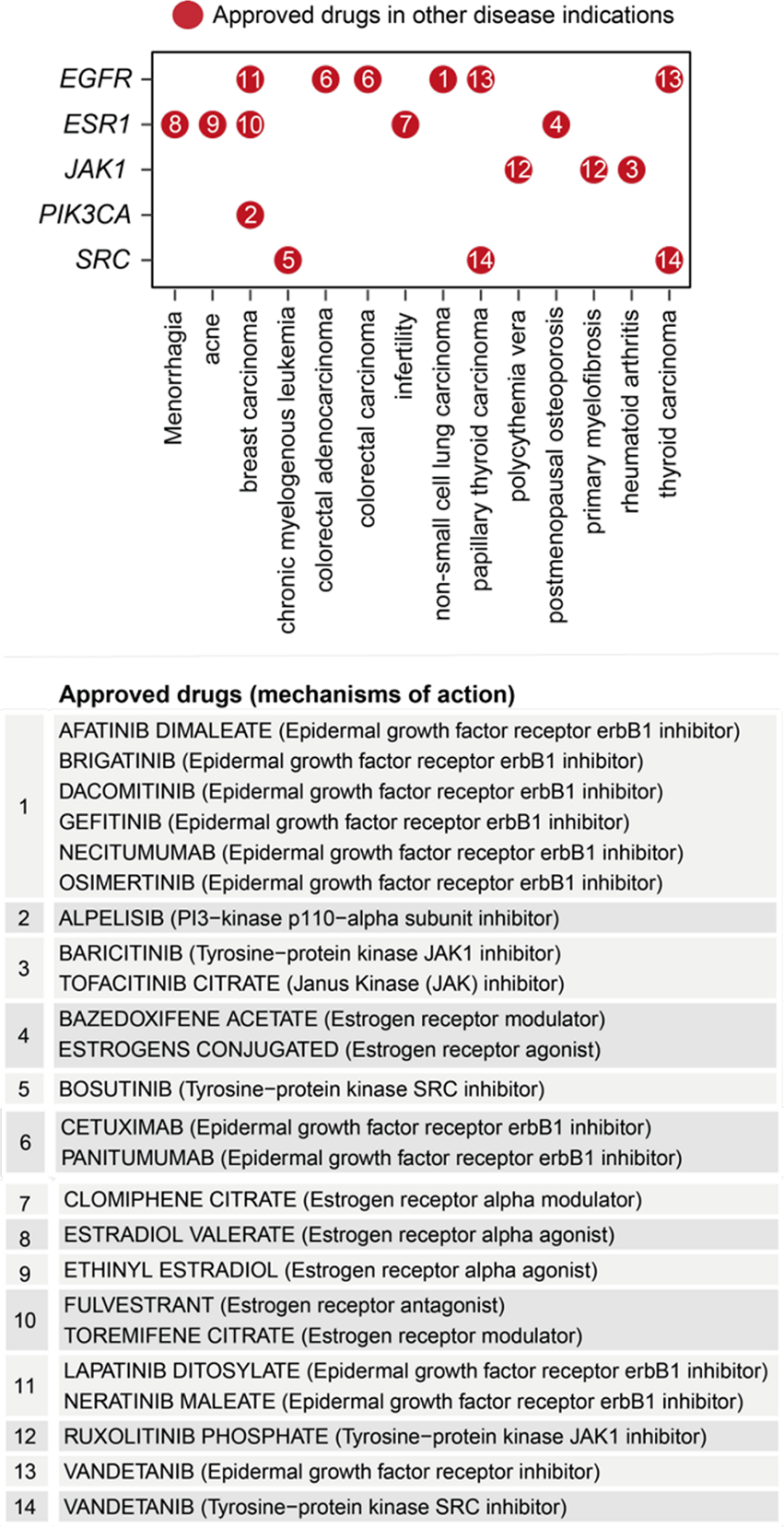
Repurposing analysis of licensed medications based on pathway crosstalk genes. Dot plot shows approved drug target genes (y-axis) and disease indications (x-axis), with dots indexed in number and referenced beneath showing the information on drugs and mechanisms of action.

Removing/attacking a node critical for the crosstalk would result in large numbers of disconnected nodes. We found that the crosstalk identified above was very robust to node removal. The maximal effect achieved by any individual node removal was as low as 10% network disconnection (that is, removing *AKT1* gene; **Figure 6C**). The identification of *AKT1*, or more generally, genes in the PI3K/AKT/mTOR pathway (including *MTOR*, *PIK3CA*, and *PIK3R1* in addition to *ATK1*; see also **Figure 5**), is in line with current interests targeting this pathway in endometriosis (65). This motivated us to further perform combinatorial attack analysis in the context of *AKT1*. The 2-node maximal effect was observed when removing another approved drug target, *ESR1*, *EGFR*, *JAK1*, *PIK3CA* or *SRC* (**Figure 6C**). Therapeutic agents targeting *ESR1* are now under phase 3 clinical trials in endometriosis (66), and thus, we further performed attack analysis in the context of *AKT1* + *ESR1*, with maximal network disconnection (~16%) achieved by 3-node removal (**Figure 6C**). These findings supported the usefulness of our identified crosstalk genes for drug repurposing (**Figure 7**). For example, estrogen receptor modulators/agonists targeting *ESR1* are now used in the clinic; this includes bazedoxifene acetate and conjugated estrogens for preventing postmenopausal osteoporosis (related to oestrogen deficiency) (67).

### Cross-Disease Prioritization Map Between Endometriosis and Immune Diseases Identifies Shared and Distinct Target Gene Clusters

**Figure 8:**
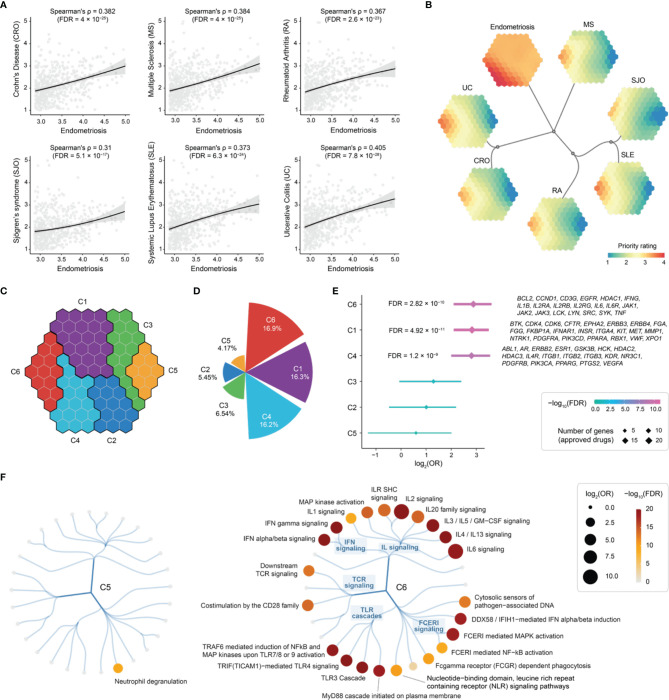
Cross-disease prioritization map between endometriosis and immune-mediated diseases identifies shared and distinct target gene clusters. **(A)** Scatter plots showing priority rating between endometriosis (x-axis) and immune diseases (each indicated in y-axis). Spearman’s rank correlation calculated, with the significance level (FDR) accounting for multiple tests. Such correlation was calculated based on the top 5% prioritized genes (n = 689) in endometriosis. **(B)** Prioritization map. Learned using a supra-hexagonal map to compare prioritizations between endometriosis and immune diseases. Each map illustrates a disease-specific gene prioritization profile, while consensus neighbor-joining tree captures the similarity of inter-disease prioritization profiles. **(C)** Target gene clusters. The prioritization map divided into 6 clusters (C1-C6), each covering continuous hexagons as color-coded. **(D)** Polar bar summarizing the percentage of approved drug targets per cluster. **(E)** Forest plot of approved drug target enrichments. **(F)** Pathway enrichments for C5 (left) and C6 (right). Reactome immune-related pathways are organized circularly, with each dot representing a pathway, sized by odds ratio (OR) and colored by FDR. FCER1, Fc epsilon receptor 1; GM-CSF, granulocyte-macrophage colony-stimulating factor; IFIH1, interferon induced with helicase C domain 1; IFN, interferon; IL, interleukin; ILR, interleukin receptor; SHC, Src homology 2-domain-containing; TCR, T-cell receptor; TLR, toll-like receptor.

A systematic review has confirmed that endometriosis has statistically significant associations with immune-mediated diseases (CRO, MS, RA, SJO, SLE, and UC) (8). Hereinafter, we explored such relationships based on the top 5% prioritized genes in endometriosis. We indeed observed significant correlations of priority ratings between endometriosis and immune diseases (**Figure 8A**). To further capture such correlations, we next employed a supra-hexagonal map (54) for cross-disease comparisons (**Figure 8B**, **Figure S2** and **Table S5**). Each presentation illustrates a disease-specific prioritization profile (in which hexagons are color-coded to show priority ratings for genes thereof), while consensus neighbor-joining tree captures the (dis)similarity of prioritization profiles between diseases. The observed relationships are consistent with therapeutic/phenotypic similarity. For example, inflammatory bowel diseases (CRO and UC) are grouped together, and inflammatory systemic diseases (SJO, SLE, and RA) stay closer.

To systematically identify target genes that are shared between diseases and are unique to specific diseases, we grouped nearby hexagons and identified 6 target clusters (C1-C6), each containing genes with similar prioritization patterns (**Figure 8C** and **Table S5**). Among these, C6 was highly rated in all diseases analyzed, displaying the highest percentage of approved therapeutics (**Figure 8D**). Notably, C5 was highly rated in endometriosis only and contained a relatively low proportion of approved drug genes (**Figure 8D**), indicative of being under-explored. In agreement with this, we found the highest degree of support from clinical evidence (approved drugs) for genes in C6, and no enrichment was observed for genes in C5 (**Figure 8E**).

Next, we characterized C5 and C6 using Reactome pathways (55). We found the enrichment for genes in C6 that are engaged in all major immune system pathways, except for neutrophil degranulation that was specific to C5 (**Figure 8F**). To confirm this, we performed functional enrichment analysis using Gene Ontology (56). We found that genes in C5 are mostly functionally relevant to neutrophil degranulation and are localized in the secretory granule lumen (**Figure S3A**). Using Mammalian Phenotype Ontology (57), we also observed phenotypic enrichments for genes in C5; these genes, when knocked out, tended to cause lethality phenotypes (**Figure S3B**).

### Exploring Repurposing Opportunities Based on Shared Target Genes

**Figure 9:**
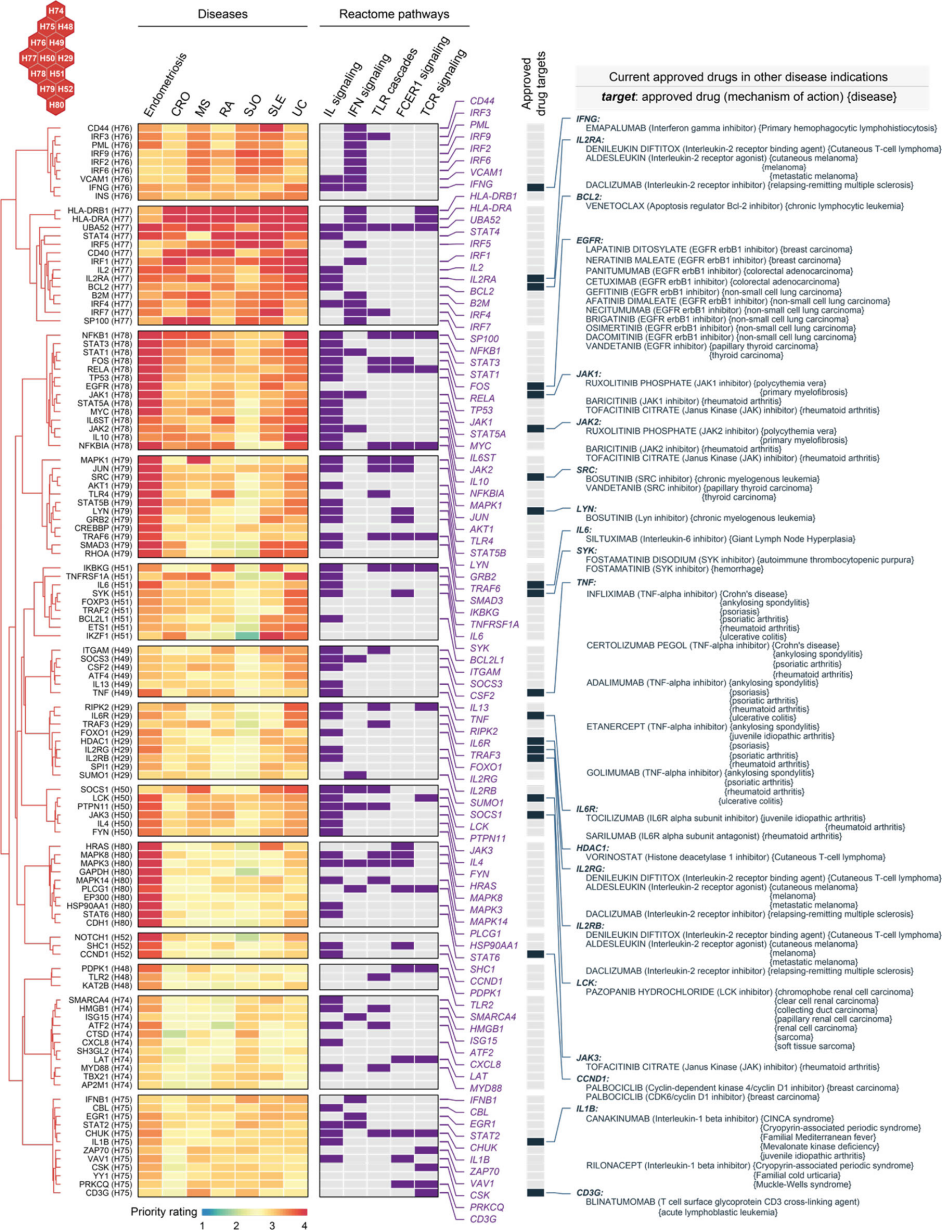
Repurposing evidence for immunomodulatory drugs based on shared target genes in the cluster C6. Heatmap illustrates target genes in C6 prioritized across 7 diseases, with annotations to interleukin (IL), interferon (IFN), toll-like receptor (TLR), Fc epsilon receptor 1 (FCER1), and T-cell receptor (TCR) signalings. Also annotated is the information on approved drug targets, repurposed drugs, mechanisms of action, and disease indications.

Genes in C6 were highly prioritized across diseases (**Figure 8**). Based on these genes, we explored repurposing opportunities *via* a heatmap-like illustration (**Figure 9**). In addition to priority ratings, this illustration collectively showed how these genes are particulate in immune system pathways, including cytokine signaling (interferon signaling and interleukin signaling), innate immunity (toll-like receptor and Fc epsilon receptor 1) and adaptive immunity (T-cell receptor). We identified 20 approved drug targets that are mostly immune-related. These genes provide opportunities for licensed immunomodulatory drugs that could be repurposed for the potential use in endometriosis, particularly disease-modifying anti-rheumatic drugs (DMARDs). They include biological DMARDs (such as inhibitors of TNF, IL6, and IL6R) and targeted synthetic DMARDs (such as kinase inhibitors targeting *JAK1/2/3*, *SRC*, *LYN*, *SYK*, and *LCK*). Of particular interest, *TNF* is a well-established therapeutic target for chronic inflammatory diseases (68). We suggest that drugs targeting this gene should be studied in cohort-scale clinical trials for repurposing in endometriosis, such as infliximab (known as anti-TNF biologics).

### Exploring Tractable Evidence for Neutrophil Degranulation Target Genes Specific to Endometriosis

**Figure 10:**
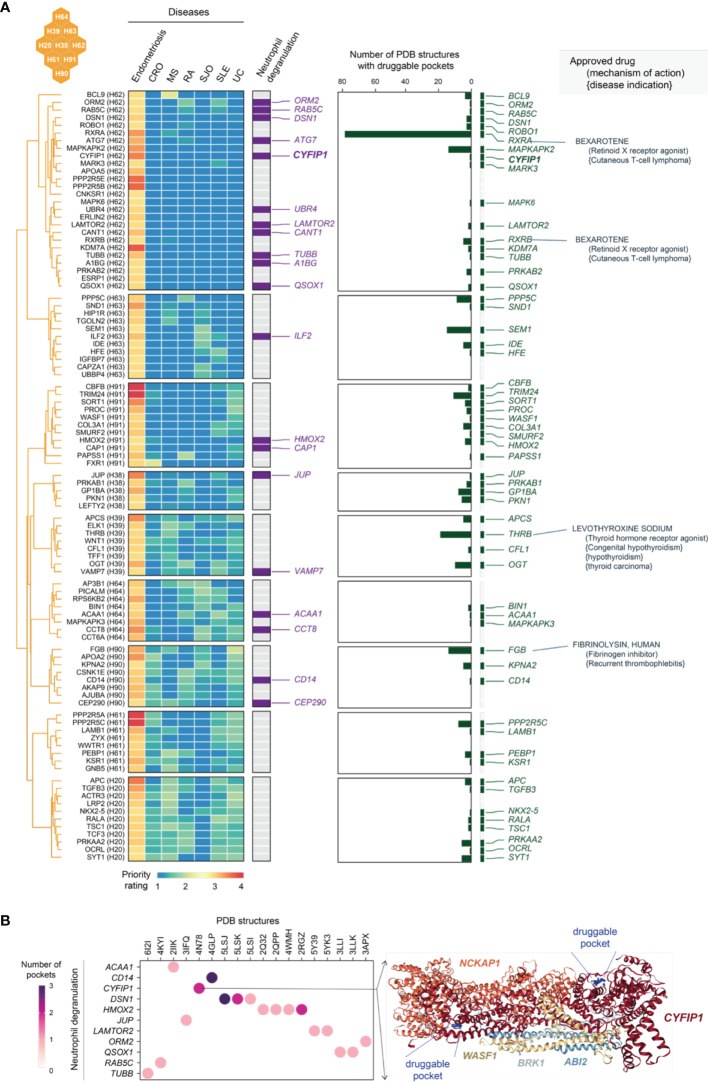
Tractable evidence for targeting neutrophil degranulation based on genes prioritized specific to endometriosis in the cluster C5. **(A)** Heatmap illustrates target genes in C5 prioritized across 7 diseases, together with the information on functional relevance to neutrophil degranulation, the tractability (the number of druggable pockets), and therapeutic approval (approved drug targets, repurposed drugs, mechanisms of action, and disease indications). **(B)** Druggable pockets for genes prioritised specific to endometriosis in C5. Dot plot shows 11 tractable genes involved in neutrophil degranulation (y-axis) and their PDB known protein structures (x-axis). Color-coded is the number of druggable pockets predicted based on the PDB structure. On the right is an exemplar illustrating an experimentally resolved WAVE regulatory complex coded as ‘4N78’ in PDB. This structure consists of 5 genes including *CYFIP1*, where two druggable pockets are predicted (as indicated in blue balls and labelled as well).

As shown in the heatmap visualization in **Figure 10A**, genes in C5 were specifically prioritized in endometriosis and characterized by neutrophil degranulation. Neutrophils are highly versatile and plastic. Plastic neutrophils are rendered through degranulation, in which cytoplasmic granules are mobilized and fused with the plasma membrane (69). Accumulating evidence supports the tumour metastatic spread model in which neutrophils mediate the migration of cancer cells to distant sites (70). Interestingly, we found that neutrophil degranulation genes in C5 are almost all linked to cancer; for example, those involved in breast cancer are the genes *ATG7* (71), *CAP1* (72), *CCT8* (73), *CD14* (74), *CYFIP1* (75), and *QSOX1* (76). Notably, neutrophil degranulation genes have been reported to be associated with endometriosis, such as *A1BG* (a diagnostic marker for stage II, III and IV endometriosis) (77) and *ATG7* (an autophagy gene in ovarian endometriosis) (78). Finally, we explored the tractable evidence; a tractable gene was defined if its known protein structures were predicted to contain druggable pockets (**Figure 10B**). We suggest that the tractable genes involved in neutrophil degranulation are of particular interest to inform future clinical studies.

## Discussion

Our organismal systems (mainly immune, endocrine, nervous, and reproductive) are evolutionarily conserved to maintain homeostasis. If these systems fail to coordinately respond to invaders or ectopic hazards, autoimmunity or other forms of systemic inflammation might occur. If such maladaptation persists, it may eventually lead to chronic inflammatory systemic diseases (64). We have found that genetic target genes prioritized in endometriosis are significantly enriched in a wide range of organismal system pathways (**Figure 6**) and molecular hallmarks (**Figure 5**). These findings strongly support the increasing recognition of endometriosis as an inflammatory systemic disease.

Our identification of target genes shared with immune diseases suggests drug repurposing opportunities (**Figure 9**). Drug repurposing is a strategy that repositions existing effective drugs with acceptable safety from original indications (such as immune diseases) to new indications (here, endometriosis). Immunomodulatory targets and DMARDs are rich in numbers and have been well-studied in therapeutic targeting. In the next steps, DMARDs represent a wish list that should be taken forward for validation, for example, to assess efficacy on perturbation in patient-derived cell assays (53). Given that multiple cell lineages might be involved in endometriosis (**Figure 4**), we utilized single-cell RNA-seq datasets of endometriosis (49) to examine the expression of shared target genes (listed in **Figure 9**). We found that these genes were expressed in one or more cell clusters that were assigned to patients with endometriosis (**Figure S4A**). Using genes specifically expressed in fibroblasts of ectopic endometrium (as compared to fibroblasts of normal endometrium) (49), we showed that these fibroblast-specific genes tended to be highly prioritized (**Figure S4B**), supporting the importance of fibroblasts in endometriosis.

We know that neutrophils are highly plastic and can mediate the transport of cancer cells into distant sites; plasticity and metastasis-like spread are both enabled by granule mobilization and exocytosis (known as ‘degranulation’) (69, 70). Systemic circulating neutrophils from endometriosis patients display distinct expression profiles when compared to neutrophils from healthy controls, and the lesion microenvironment contains factors such as IL-8 that promote recruitment of neutrophils (79). The identification of endometriosis-specific targets essential for neutrophil degranulation (**Figure 10**) not only brings forward new ideas for therapies targeting neutrophil degranulation, but also provides important clues about how endometriosis might spread to distant tissues or organs, causing inflammatory-like microenvironments. Our findings expand the existing theories that are proposed to explain the cause of endometriosis (the origin of disease), including: (i) Sampson’s retrograde menstruation (or the implantation theory), stating that at menses, endometrial cells reflux through the fallopian tubes and invade pelvic structures, where coordinated growth of nerves and blood vessels (known as ‘neuroangiogenesis’) may occur and elicit an inflammatory response, fibrotic scarring and pain; (ii) the coelomic metaplasia theory (80), postulating that mesothelial cells may transform into ectopic endometrial tissue through metaplastic transition; and (iii) stem cell trafficking, particularly endometrial mesenchymal stem/stromal cells (81, 82) and bone marrow-derived stem cells (83). We anticipate that consolidating mixed evidence to unify all findings (including ours) might be the most parsimonious explanation for this inflammatory systemic disease.

We have also used gene expression signatures (50) involving two endometriosis stages (I/II and III/IV) at two menstrual cycle phases (proliferative and secretory) to examine the highly prioritized target gene expression (**Figure S5A**). Target set enrichment analysis revealed the enrichment of gene signatures in stage I/II endometriosis (early disease), independent of menstrual cycle phases. For stage III/IV endometriosis (late disease), the enrichment was observed at the proliferative cycle phase but not at the secretory cycle phase. These results suggested that highly prioritized target genes tended to be expressed in the early stage of disease, which is consistent with the pro-inflammatory profile in stage I/II endometriosis (50). We also found that genes highly prioritized only in endometriosis (listed in **Figure 10**) were mostly expressed in stage I/II disease (relative to the healthy counterpart; **Figure S5B**).

In summary, we have shown that multi-layered genomic datasets (including new information on genetic susceptability loci identified in GWAS on endometriosis, regulatory genomics, and protein interactome) can be harnessed with our prioritization approach to generate an atlas of genetic target prioritizations in endometriosis (**Figures 1** **-** **3**). This atlas has enhanced our understanding of endometriosis as an inflammatory systemic disease and has also expanded the existing theories on the origin of disease. More importantly, we have discovered target candidates that are specific to and tractable for disease. Genomic insights and therapeutic candidates arising from this study may change our perspectives on endometriosis and our strategies for coping with disease, envigorating further research and drug repurposing.

## Data Availability Statement

The original contributions presented in the study are included in the article/**Supplementary Material**. All results, including an atlas of genetic targets and repurposed drugs in endometriosis, are publicly available at https://23verse.github.io/end for download and exploration. Further inquiries can be directed to the corresponding author.

## Author Contributions

CB analyzed data, contributed to data curation, wrote and revised the manuscript. HW contributed to data curation and interpretation. HF conceived and supervized the project, curated and analyzed data, wrote and revised the manuscript. All authors contributed to the article and approved the submitted version.

## Funding

This work is funded by National Natural Science Foundation of China (32170663), Shanghai Pujiang Program (21PJ1409600), Innovative Research Team of High-Level Local Universities in Shanghai, and Program for Professor of Special Appointment (Eastern Scholar) at Shanghai Institutions of Higher Learning (awarded to HF).

## Conflict of Interest

The authors declare that the research was conducted in the absence of any commercial or financial relationships that could be construed as a potential conflict of interest.

## Publisher’s Note

All claims expressed in this article are solely those of the authors and do not necessarily represent those of their affiliated organizations, or those of the publisher, the editors and the reviewers. Any product that may be evaluated in this article, or claim that may be made by its manufacturer, is not guaranteed or endorsed by the publisher.
