# Supplementary material for: Genomic Evidence Supports the Recognition of Endometriosis as an Inflammatory Systemic Disease and Reveals Disease-Specific Therapeutic Potentials of Targeting Neutrophil Degranulation
Source: Front Immunol. 2022 Mar 23;13:758440. doi: 10.3389/fimmu.2022.758440 (PMC8983833; doi:10.3389/fimmu.2022.758440)
Supplement: Supplementary file 1 [file DataSheet_1.pdf]

Supplementary Material

1 Supplementary Figures

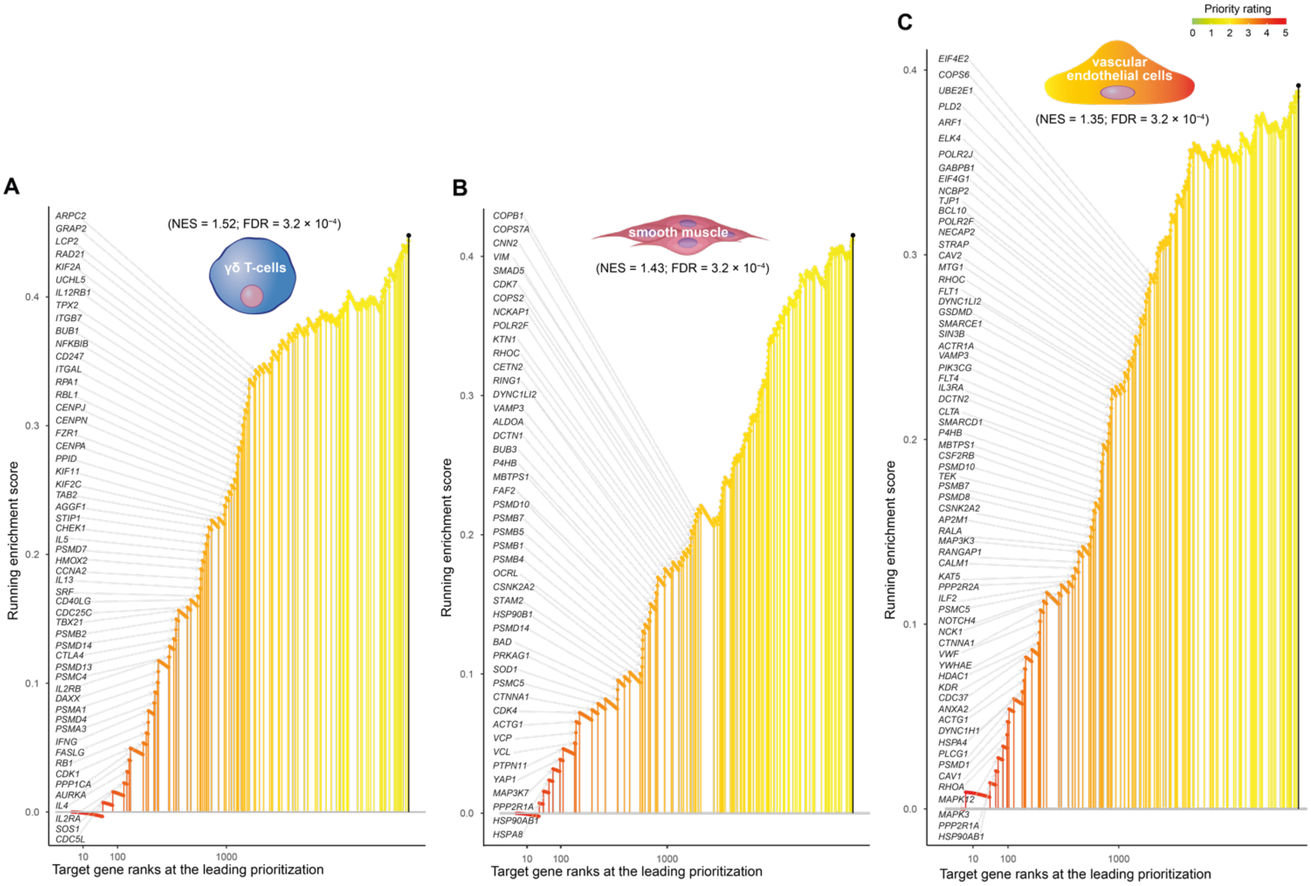

Supplementary Figure 1 | Cellular basis of therapeutic targeting in endometriosis. (**A-C**) Leading prioritization plots for the top enriched cell types 'gamma delta T-cells' (**A**), 'smooth muscle' (**B**) and 'vascular endothelial cells' (**C**). Each plot illustrates target ranks for cell-type-specific genes found at the leading prioritization (indicated in vertical lines, colored by priority rating, and also labelled if prioritized at the top 10% for simplicity).

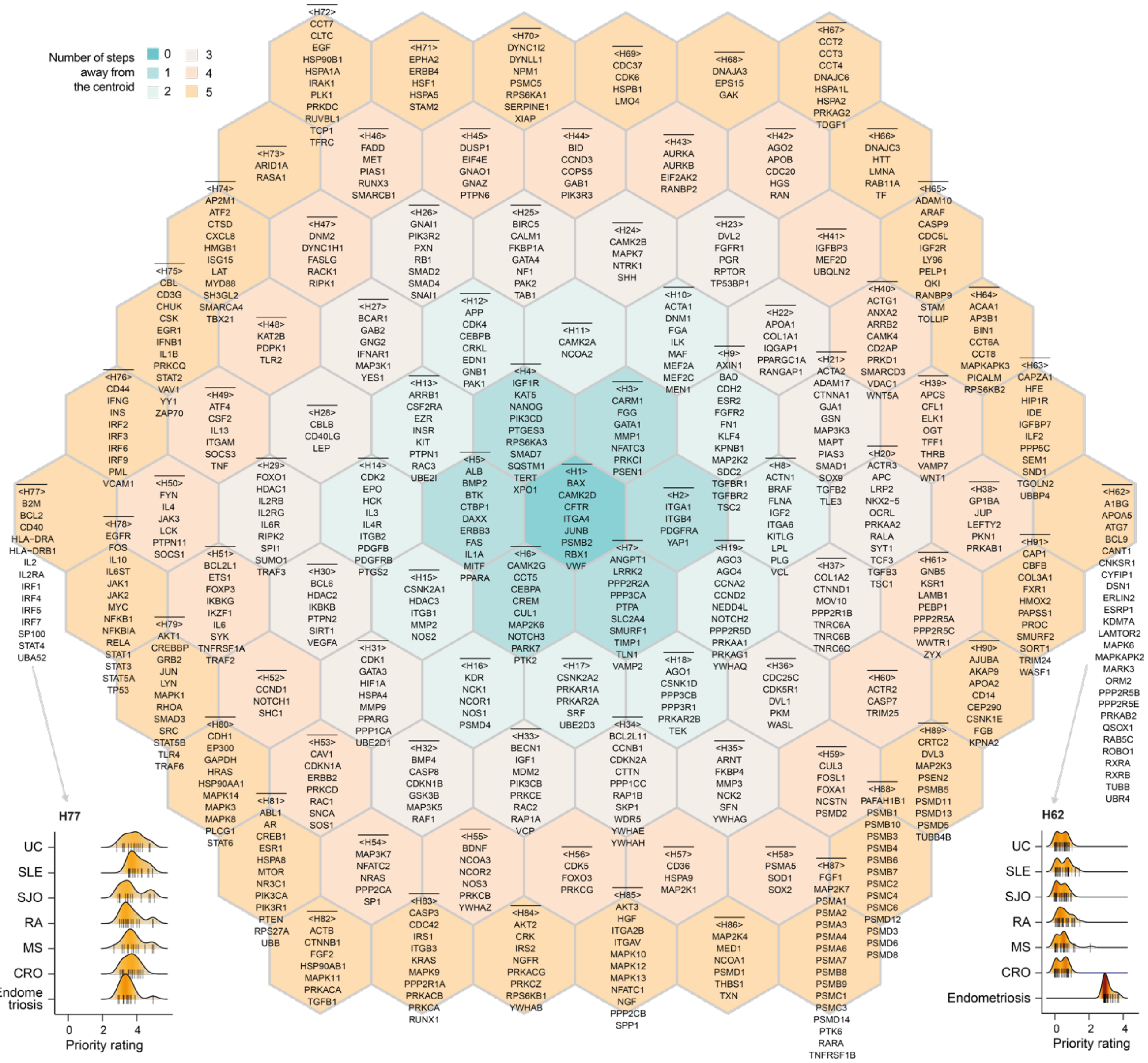

Supplemental Figure 2 | Illustrating the architecture of the supra-hexagonal map. This map consists of 91 hexagons that are indexed circularly outward (H1-H91; such circular indexing is indicated in colors). Also displayed are genes in each hexagon. Beneath are ridge plots for two hexagons, showing density of priority rating for genes in H77 (bottom-left) and in H62 (bottom-right). Notably, genes in H77 are highly rated in all diseases, while genes in H62 highly rated only in endometriosis.

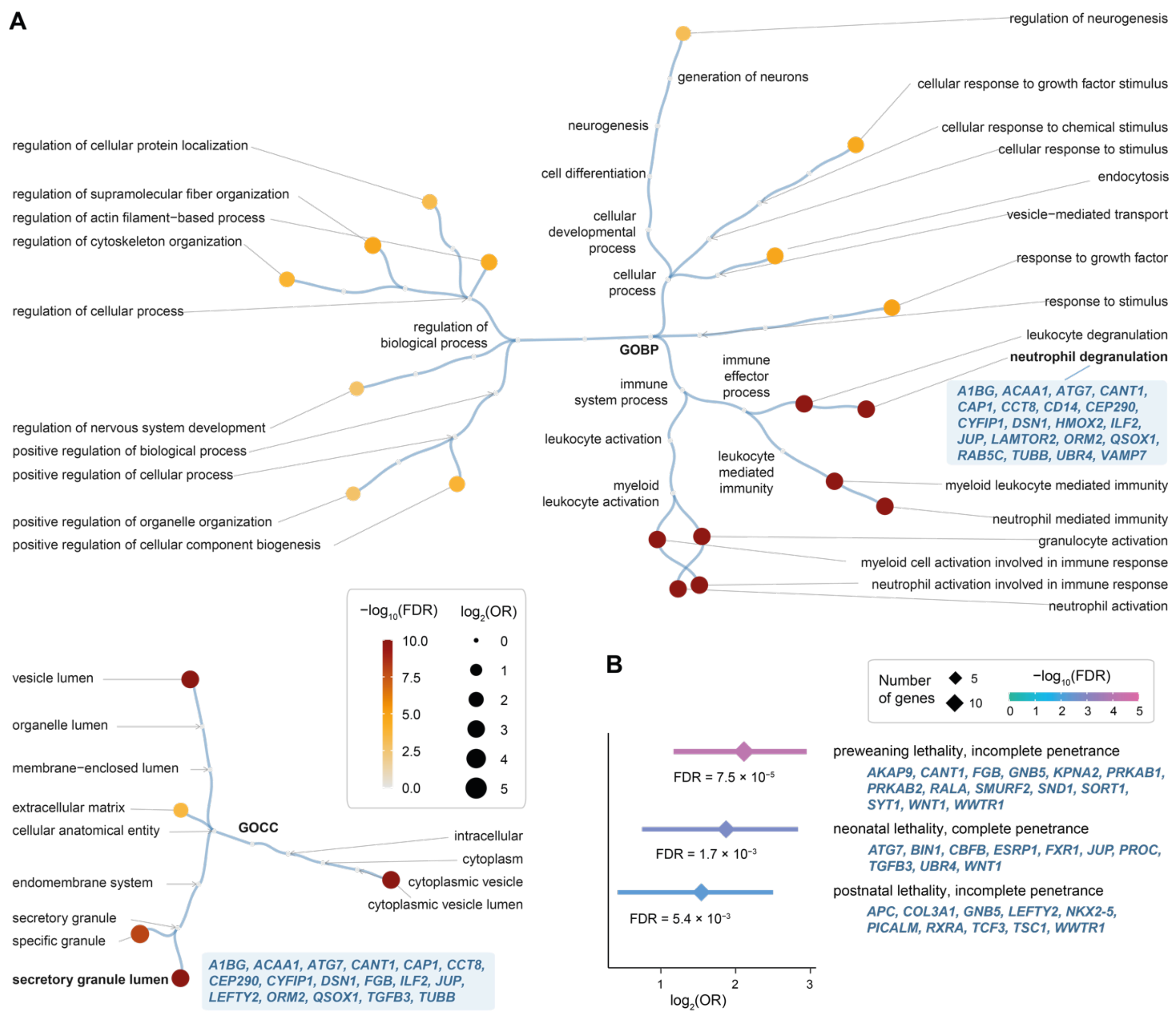

Supplementary Figure 3 | Characterization of distinct target gene cluster (C5). (**A**) Functional enrichments using Gene Ontology (GO). Each dot represents a GO term, sized by odds ratio (OR) and colored by FDR. GOBP, Gene Ontology Biological Process; GOMF, Gene Ontology Molecular Function. (**B**) Forest plot of mouse phenotype enrichments.

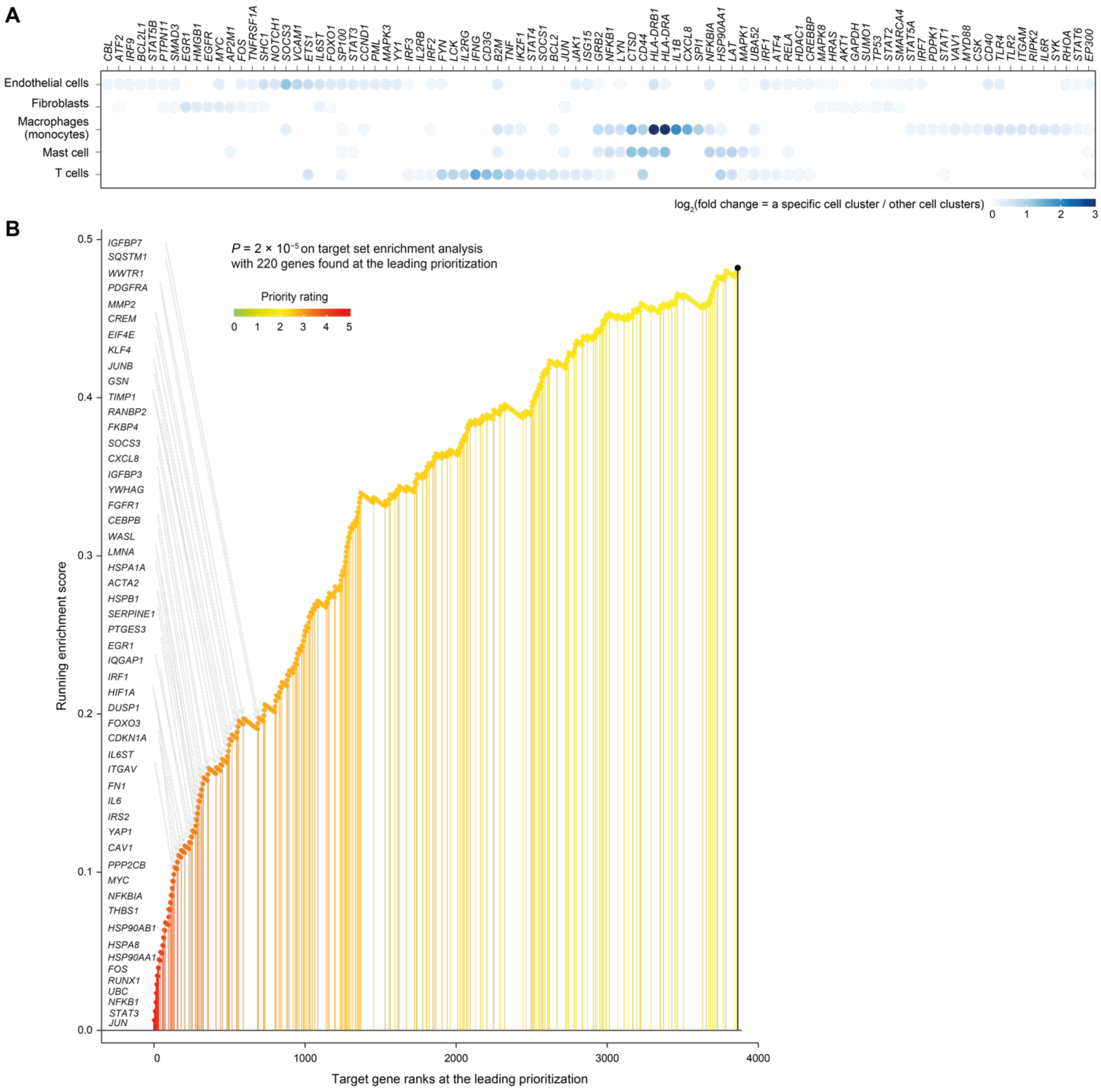

Supplementary Figure 4 | Analysis using single-cell RNA-seq datasets of endometriosis. (**A**) Dot plot showing the expression of shared target genes (listed in **Figure 9**; x-axis) in cell clusters assigned to patients with endometriosis (y-axis). (**B**) Leading prioritization plot for genes specifically expressed in fibroblasts of ectopic endometrium. Genes are labelled if prioritized at the top 5 % for simplicity.

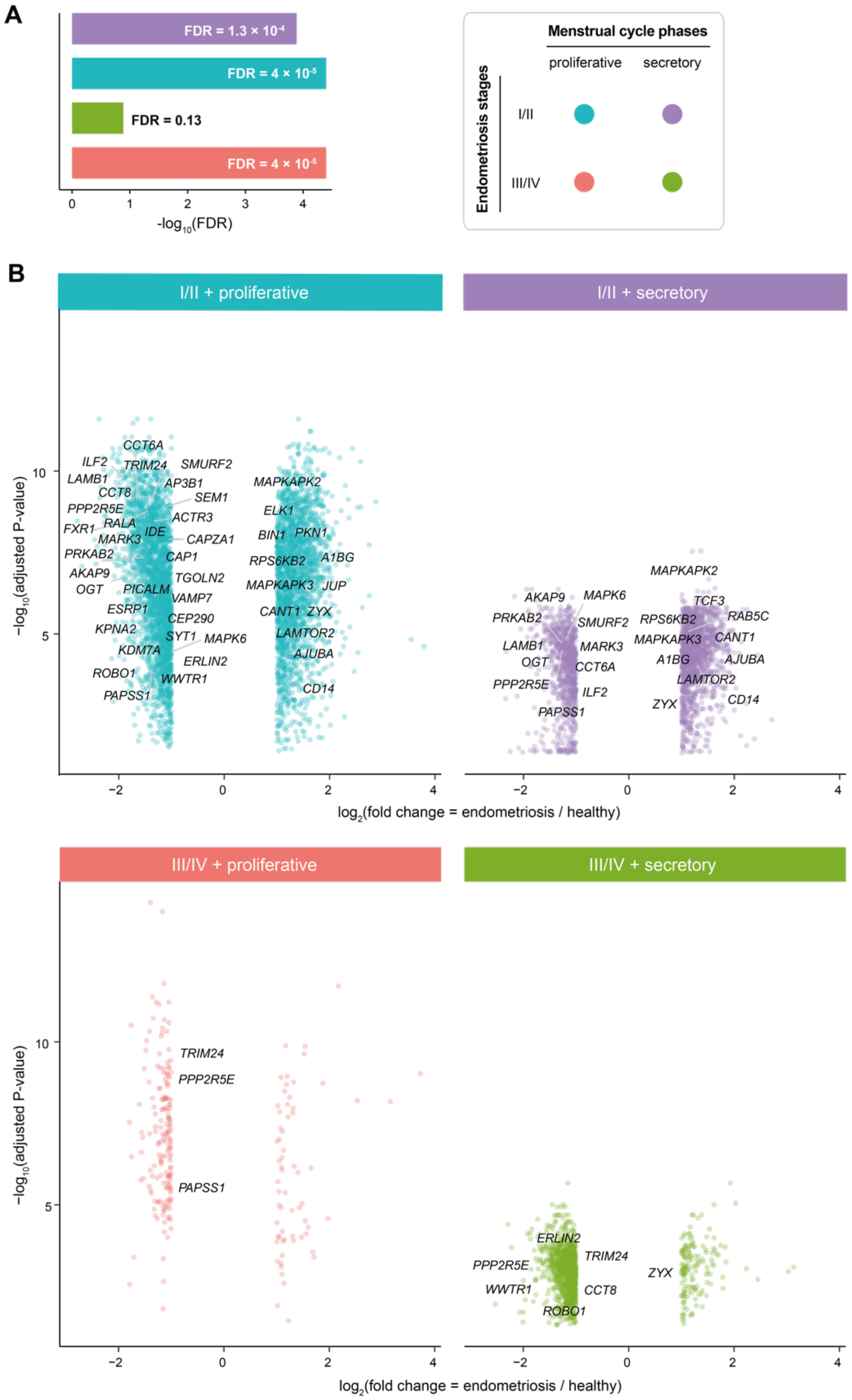

Supplementary Figure 5 | Analysis using gene expression signatures involving two endometriosis stages (I/II and III/IV) at two menstrual cycle phases (proliferative and secretory). (**A**) Bar plot showing signature enrichments when target set enrichment analysis applied to all prioritized target genes in endometriosis. (**B**) Volcano plot showing gene expression signatures, with genes highly prioritized only in endometriosis (listed in **Figure 10**) labelled.

2 Supplementary Tables

Supplementary Table S1. Prioritized list of target gens in endometriosis.

Supplementary Table S2. Cell type enrichments.

Supplementary Table S3. Molecular hallmark enrichments.

Supplementary Table S4. Genes in the crosstalk.

Supplementary Table S5. Target gene clusters.
